# Service evaluation of weight outcomes as a function of initial BMI in 34,271 adults referred to a primary care/commercial weight management partnership scheme

**DOI:** 10.1186/1756-0500-6-161

**Published:** 2013-04-24

**Authors:** Richard James Stubbs, David Johnathan Brogelli, Jenny Barber, Carolyn Pallister, Stephen Whybrow, Amanda Avery, Jacquie Lavin

**Affiliations:** 1 Nutrition and Research Department, Slimming World, Clover Nook Road, Somercotes, Alfreton, Derbyshire, DE55 4RF, UK; 2 University of Derby, Kedleston road, Derby, Derbyshire, DE22 1GB, UK; 3 Public Health Nutrition Research Group, University of Aberdeen, Aberdeen, AB25 2ZD, UK

**Keywords:** Obesity, Treatment, Weight management, Body mass index, Weight outcomes

## Abstract

**Background:**

It is not clear if behaviour change programmes are more or less effective for weight management in people with high BMIs than for those who are moderately overweight. An earlier service evaluation reported on the rate and extent of weight loss in a primary care/commercial weight management organisation partnership scheme, in 34,271 patients were referred by their health care professionals to a UK commercial weight management organisation, Slimming World for 12 weekly sessions. This project updated that service evaluation by examining weight loss outcomes as a function of initial BMI in the same 34,271 patients.

**Findings:**

Patients referred to the scheme (n = 34,271) were categorised by BMI groups <30 kg/m^2^, 30-34.9 kg/m^2^, 35-39.9 kg/m^2^ and to ≥ 40 kg/m^2^. Mean weight losses after 12 weekly sessions were 2.9, 3.6, 4.1, and 4.8 kg for each BMI category respectively. Regression analysis showed that after adjusting for age and gender, relative to the <30 kg/m^2^ group, absolute weight losses were 0.8, 1.4 and 2.4 kg more for the 30-34.9 kg/m^2^, 35-39.9 kg/m^2^ and to ≥ 40 kg/m^2^ groups, respectively (all p<0.001). Percent weight loss was similar in each BMI category: 3.7%, 4.0%, 4.0% and 3.9%, respectively (p<0.001).

**Conclusions:**

This service evaluation demonstrates that 12 week referral to a commercial organisation is as effective for people with high BMIs as for those who are moderately overweight.

## Background

In a previous paper we have examined the rate and extent of weight loss in a primary care/commercial weight management organisation (CWMO) partnership scheme (called Slimming on Referral). In that paper 34,271 patients were referred by their health care professionals to a UK commercial weight management organisation, Slimming World, for 12 weekly sessions [1]. Data were reported for the whole population, for completers (those who attended 10 of 12 sessions) versus non-completers, and for men and women [1]. There is now a growing body of data suggesting that commercial diet and lifestyle programmes are effective in the general population [1-8]. However, it is not clear from current published studies whether lifestyle interventions are as effective in patients with higher BMIs than for those who are moderately overweight [1,2].

The purpose of the current analysis was to examine the effectiveness of a primary care/CWMO partnership scheme in patients of different BMI categories. Data were collected from participants in the slimming on referral scheme between May 2004 and November 2009, who had time to finish their full 12-week referral. This resulted in the inclusion of 38,614 patients who were referred from within 77 Primary care Trusts or NHS Trusts, for whom data on weight, height, age and gender were collected. Of these there were 2,625 cases where the data for one or more of the 12 referral vouchers issued per participant was unclear or could not be resolved (spoiled vouchers, illegible writing etc.) and 1,718 cases where the participant completed the scheme outside of the 14 (12 plus 2) week time window, due to circumstances such as bereavement or illness. This left 34,271 in the present study. 80 participants were included in the database but there was no data for their date of birth. Age data are reported excluding these subjects. Some participants went on to self-fund further attendance following the initial 12-week referral and others were offered subsequent 12-week referral packages from their health care team. Results for the latter are reported elsewhere [9]. The data in this analysis covers the initial 12-week sessions of the referral scheme.

Data for this service evaluation was collected as part of routine data collection within the referral programme. At the point of referral patients’ gender, date of birth and height were recorded by the health professional. When the patient enrolled at the weight management group (week 1 of the referral), their start weight and date were recorded. Each week the patient returned to group their weight change was recorded along with date of attendance. The same calibrated scales were used each week at a given group to record weight and weight change. The collected data were sent to the research team for input into the referral database.

This work is categorised as a service evaluation under the Ad Hoc Advisory Group on the Operation of NHS Research Ethics Committees, guidelines (2006). Existing data were anonymised and analysed as an intervention in use only to ask the question "What standard does this service achieve?"

Data were extracted from the referral database, and subjected to a number of parameter checks for outliers, and anomalous data entry. Anomalies were checked against the raw source data to resolve any issues that arose. From the raw data collected, start BMI, end BMI, BMI change, weight change and percent weight change were calculated. There was considerable variability in the number of sessions attended, ranging from 1–12 weeks. Mean attendance was 8.9 of 12 sessions.

The end weight was calculated based on the members’ last attendance at group during the referral period using the Last Observation Carried Forward approach [10].

For the current analysis weight loss outcomes were analysed by the BMI categories <30 kg/m^2^, 30–34.9 kg/m^2^, 35–39.9 kg/m^2^ and ≥40 kg/m^2^. The effects of different factors on weight loss between BMI categories were assessed, by fitting linear models and examining the significance of fitted terms in these models, through regression and analysis of variance. All analysis was performed using the GENSTAT 5 statistical program (Genstat 5 Rothampstead Experimental Station, Harpenden, UK). Results are expressed as mean (SD).

## Findings

The physical characteristics and weight outcomes by BMI category are given in Table 1. Eleven percent had a start BMI <30 kg/m^2^, 34% between 30–34.9 kg/m^2^, 29% between 35–39.9 kg/m^2^ and 26% had a start BMI ≥40 kg/m^2^. Weight, end BMI, BMI change and absolute weight loss all increased with increasing BMI category (all p<0.001). Absolute weight losses over the 12 week study period were 2.9, 3.6, 4.1, and 4.8 kg for the BMI categories <30 kg/m^2^, 30–34.9 kg/m^2^, 35–39.9 kg/m^2^ and ≥40 kg/m^2^, respectively. Regression analysis showed that after adjusting for age and gender, relative to the <30 kg/m^2^ group, absolute weight losses were 0.8, 1.4 and 2.4 kg more for the 30–34.9 kg/m^2^, 35–39.9 kg/m^2^ and ≥40 kg/m^2^, groups, respectively (all p<0.001). Percent weight loss was similar in each BMI category at 3.7, 4.0, 4.0 and 3.9% for BMI categories <30 kg/m^2^, 30–34.9 kg/m^2^, 35–39.9 kg/m^2^ and ≥40 kg/m^2^. Regression analysis showed that after adjusting for age and gender, relative to the <30 kg/m^2^ group, percent weight losses were 0.3, 0.3 and 0.2% greater for the 30–34.9 kg/m^2^, 35–39.9 kg/m^2^ and ≥40 kg/m^2^ groups, respectively (all p<0.001). For each BMI category those achieving 5% weight loss were 33, 37, 36 and 36%, respectively (main effect, p=0.006). Regression analysis showed that significant differences occurred between the BMI categories <30 kg/m^2^ and 30–34.9 kg/m^2^ (p=0.028). The percentage of those losing 10% in their first 12 sessions by BMI category were 6, 6, 6 and 5% respectively (main effect, p=0.011). Specific group differences were not significant in regression comparisons. There was no significant difference in the number of weeks attended as a function of BMI category (p=0.905).

**Table 1:** Patient characteristics at week 1 of the referral scheme and weight change by start BMI category

	**BMI < 30 kg/m**^**2**^		**BMI 30**–**34.9 kg/m**^**2**^		**BMI 35**–**39.9 kg/m**^**2**^		**BMI ≥ 40 kg/m**^**2**^			
	**Total n=3697**		**Total n=11759**		**Total n=9902**		**Total n=8913**			
	**Mean**	**SD**	**Mean**	**SD**	**Mean**	**SD**	**Mean**	**SD**	**f-value**	**p-value**
**Height (m)**	1.65	0.08	1.64	0.08	1.64	0.08	1.64	0.08	48.60	<0.001
**Weight (kg)**	76.8	8.0	88.0	9.6	100.8	10.9	122.3	18.6	19885.38	<0.001
**Age (years)**	48.1	15.0	48.1	14.8	47.2	14.6	45.8	13.4	64.43	<0.001
**Weight change (kg)**	−2.9	2.8	−3.6	3.2	−4.1	3.7	−4.8	4.4	346.58	<0.001
**Percent weight change**	−3.7	3.6	−4.0	3.6	−4.0	3.6	−3.9	3.5	7.06	<0.001
**Weeks attended**	8.7	3.6	8.9	3.5	8.8	3.6	8.9	3.5	0.19	0.905
**Start BMI (kg/m**^**2**^**)**	28.2	1.4	32.5	1.4	37.3	1.4	45.4	5.2	44536.13	<0.001
**End BMI (kg/m**^**2**^**)**	27.2	1.7	31.2	1.8	35.8	1.9	43.6	5.3	37989.02	<0.001
**BMI change (kg/m**^**2**^**)**	−1.1	1.0	−1.3	1.2	−1.5	1.3	−1.8	1.6	387.75	<0.001
**Proportion achieving 5% Weight loss**	33.4%		36.6%		36.4%		35.8%		4.10	0.006
**Proportion achieving 10% Weight loss**	5.5%		6.1%		6.2%		5.4%		3.69	0.011

The current analysis illustrates that percent weight loss was consistent across the BMI range and that those with BMIs ≥40 (who represented 26% of participants referred from primary care) lost a similar percentage of their initial body weight as other participants at a lower BMI. In the present service evaluation, participants followed a group support programme and dietary plan, which is structured around *ad libitum* intake of low energy dense foods, principles of energy balance and appetite regulation to reduce energy intake, with additional guidance to ensure a balanced diet [11]. The present data set suggests that participants with a BMI ≥40 can achieve higher absolute and similar percent weight losses to their counterparts at lower BMIs when following a low energy density dietary plan, *ad libitum*.

Average start BMI of the study population was in the range that would be recommended for more intensive interventions such as pharmacotherapy and for a significant percentage, bariatric surgery [12,13]. Start BMI averaged 36.8 kg/m^2^ and 26% of this referral population had a BMI ≥40 kg/m^2^. This suggests that lifestyle interventions can work in populations with BMIs that are normally recommended to receive secondary or tertiary care.

As this was a service evaluation, it was limited by the absence of a control group and the fact that the results were based upon those people who joined a group, rather than intention to treat. The study only observed weight changes over 12 weekly sessions and there was no longer-term follow up. Key strengths were that the referral programme evaluation assessed the effectiveness of the programme as it runs in real life, the sample size was large and conducted in members of the general public aiming to control their weight in their everyday lives.

## Conclusion

Referral to a commercial organisation is as effective for people with high BMIs as for those who are less overweight and attendances were similar between BMI categories.

## Abbreviations

CWMO: Commercial weight management organisation; SD: Standard Deviation; BMI: Body Mass Index; NHS: National Health Service.

## Competing interests

RJS, DJB, JB, CP, AA and JL work for Slimming World. SW was supported by Slimming World for the analysis and preparation of this manuscript.

## Authors’ contributions

JS, DB, JB, CP and SW collated the data, JS, CP, AA and JL were involved in the design of the study, JS and SW were involved in the statistical analysis, JS, CP and DB drafted the manuscript. All authors read and approved the final manuscript.
